# Intensification Technologies to Efficiently Extract Antioxidants from Agro-Food Residues

**DOI:** 10.3390/antiox10101568

**Published:** 2021-10-01

**Authors:** Soraya Rodríguez-Rojo

**Affiliations:** High Pressure Processes Group, Research Institute on Bioeconomy (BioEcoUVa), School of Engineering, University of Valladolid (UVa), 47011 Valladolid, Spain; soraya.rodriguez@uva.es

As is well known, there is an increasing interest in recovering phytochemicals from agricultural, forestry, and food industry residues, aiming to reduce their environmental impact and improve sustainable economic growth in the bioeconomy scheme. These phytochemicals can be employed widely in food and feed, food supplements, and cosmetics products, among others, thanks to their bioactivity properties, such as being antioxidant, antimicrobial, etc.

The recovery of bioactive compounds from vegetal matrices involves several steps, where an adequate pre-treatment and extraction are of foremost importance. Conventional solid–liquid extraction is the technique of choice at an industrial scale. Its main drawbacks are the high solvent consumption and long extraction times that may degrade thermolabile phytochemicals due to the relative high temperature required to improve mass transfer. Additionally, the fact that some bioactive molecules, i.e., polyphenols, have internal cell localizations or are bounded to cell wall polysaccharides limits the extraction yield. 

Therefore, the industrial sustainability of the recovery of phytochemicals from agro-food wastes will benefit from the implementation of intensified processes covering the use of solvents with improved properties (pressurized and supercritical fluids, deep eutectic solvents or hydrotropes), nonconventional energies (microwave, ultrasound, pulsed electric), or high static pressure. All these technologies, which are being studied at laboratory and pilot scale, aim to develop highly efficient methods for the extraction of phytochemicals according to the “Green Engineering principles”.

This Special Issue of Antioxidants brings together current research on the efficient extraction of phytochemicals, namely phenolic compounds, from food by-products or under-exploited plants with innovative and clean processes. Thanks to the recognized antioxidant, moderate antimicrobial, and anti-inflammatory properties of these compounds, they can be applied as food preservatives and, more importantly, in the prevention or treatment of numerous diseases, as shown in the 16 original research papers and 1 review included in this compilation.

The review by Isidore et al. [1] provides an overview of the main bioactive compounds in hemp, which includes phenolic compounds and terpenes, apart from the cannabinoids, together with the extraction techniques applied so far for their recovery from conventional extraction or hydro-distillation to ultrasound-assisted extraction or high-pressure technologies such as supercritical fluid extraction.

High-pressure technologies have been proved as efficient processes to produce extracts enriched in bioactive compounds and enhanced bioactivity, as proved by Bordalo and co-workers [2] from Bravo de Esmolfe discards apples, a Portuguese traditional cultivar. They used a two-step strategy to produce extracts with high antioxidant activity as shown in a 3D cell model derived from NT2 cells for neuroprotective testing. The process consisted in the extraction of apolar compounds by supercritical (SC) carbon dioxide (CO2) to enhance the extraction of phenolic compounds in a second step using carbon dioxide–ethanol mixtures at supercritical conditions. A similar approach was used by Katsinas et al. [3], who tested the antioxidant and anti-inflammatory activity of phenolic extracts from olive pomace in two human ocular surface cell lines. These extracts were produced by pressurized liquid extraction (PLE) using hydroalcoholic mixtures after extraction of lipidic compounds of the olive pomace by SC-CO2. Additionally, conventionally exhausted olive pomace was used by Gómez-Cruz et al. [4] to produce hydroxytyrosolrich extract, using only water as a solvent, with antioxidant and moderate bactericidal action against some food-borne pathogens. Huamán-Castilla and collaborators [5] also employed PLE for the efficient extraction of procyanidin monomers and oligomers from grape pomace using water and protic co-solvents. Besides their antioxidant capacity, extracts showed inhibitory activity of Type 2 Diabetes Mellitus (T2DM)-related enzymes. Mihalcea et al. [6] produced lycopene-rich oleoresins by SC-CO2 extraction from Tomato peels that were formulated in whey protein and acacia gum. This product showed interesting antiproliferative activity in HT-29 cell culture. Tadic and co-workers [7] formulated an oil-in-water cream with beneficial effect in skin using as active ingredients oil and extracts from two by-products from bilberry processing, seeds, and leaves, respectively. The seed oil, rich in ω-3 and ω-6 fatty acids, was extracted using SC-CO2. On the other hand, high hydrostatic pressure was employed by Moreira and co-workers [8] to produce phenolic extracts from winter savory, a traditional and under-utilized herb. 

Other culinary herbs were extracted with new solvents, as performed by Coscueta et al. [9], using aqueous micellar solutions to optimize the extraction of phenylethyl isothiocyanate from watercress, as this compound is a recognize antioxidant, anti-inflammatory, and chemo-preventive agent. Kowalska et al. [10] employed glycerol as a protic co-solvent to optimize the extraction of phenolic compounds and chlorophyll from peppermint and nettle leaves.

Enzymatic-assisted extraction is also an interesting approach to improve the extraction of bioactives from agro-industrial by-products, as shown in the work of Bautista-Expósito and collaborators [11]. They selected the best glycosidase activity enzyme to increase the phenolic yield from wheat bran suspensions, previously autoclaved, to produce nutraceutical extract rich in ferulic acid, with improved antioxidant and anti-inflammatory activity. The extractability of bioactive compounds could be further improved by the combination of hydrothermal treatment and high hydrostatic pressure as pre-treatments of the enzymatic extraction as shown by Martín-Diana et al. [12]. Furthermore, the extracts from wheat bran produced were microencapsulated in pea protein. Syrpas and co-workers [13] demonstrated the improved efficiency of enzymatic assisted extraction over conventional solid–liquid extraction to recover fractions with a higher yield; phenolic content, namely anthocyanins; and enhanced antioxidant activity from bilberry pomace. Likewise, López-Fernández-Sobrino et al. [14] applied enzymatic assisted extraction to increase the soluble fraction of wine lees as well as its phenolic content. These extracts showed an increased antihypertensive effect with respect to untreated lees. 

Microwaves were employed by Álvarez and co-workers [15] as pre-treatment to conventional solid–liquid extraction of polyphenols, namely anthocyanins to produce natural colorants from saffron flowers. The control of low-energy microwave pre-treatment improved the product quality in terms of polyphenols content, antioxidant activity, and allowed for the control of colour hue of the extract. 

Ben-Othman and collaborators [16] focused on the production of extracts from apple tree leaves rich in phloretin, a phenolic compound with antiviral properties. They optimized the ultrasound assisted (USAE) extraction parameters using hydroalcoholic mixtures as solvent. Furthermore, they analysed the difference in phenolic content and antioxidant activity of the extracts of leaves from seven different cultivars grown in Estonia. The USAE technology is already quite develop as shown in the work by Grillo et al. [17] that studied the kinetics of the extraction of polyphenols from grape stalks at laboratory scale to develop a flow-mode extraction scaled-up system including also downstream processing with a nanofiltration unit.

Overall, the papers included in this Special Issue illustrate examples of the important role of extraction technology for the efficient extraction of bioactive compounds maximizing the yield and antioxidant activity of the extracts while reducing the process needs (energy, solvent, time, etc.). In addition, many of the papers showed important bioactivities for the use of vegetal extracts as food preservatives, colorants in nutraceutical products, or as promising herbal medicines for the treatment and prevention of diseases as a high-value-added application. In sum, the latest trends of research to transform “waste into wealth” from agro-food by-products are presented.
